# Complication Prevention Beyond Glycaemic Control in Type 2 Diabetes: Cumulative Metabolic Exposure, Organ Vulnerability and Treatment Durability

**DOI:** 10.1002/edm2.70383

**Published:** 2026-11-03

**Authors:** Ferenc Sztanek

**Affiliations:** ^1^ Division of Metabolism, Department of Internal Medicine, Faculty of Medicine University of Debrecen Debrecen Hungary

**Keywords:** diabetes complications, treatment outcome, type 2 diabetes mellitus

## Abstract

**Introduction:**

Type 2 diabetes is a progressive multisystem disorder in which long‐term complication burden reflects more than contemporaneous glycaemic control. Age at onset, previous metabolic exposure, obesity‐related risk, organ injury, treatment safety and the capacity to sustain effective care interact across the disease course.

**Methods:**

This critical narrative review synthesised targeted PubMed/MEDLINE searches, international guidance, landmark and contemporary randomised trials, longitudinal cohorts, validated risk models and clinically influential reviews. A targeted update search was completed through 22 September 2026.

**Results:**

The evidence supports a clinically ordered approach in which immediate treatment‐related hazards are considered first, followed by established organ disease or a dominant preventable outcome, residual modifiable cardiometabolic exposure, treatment durability and the care systems required to translate evidence into sustained benefit. Historical trials established the importance of glycaemic, blood‐pressure and lipid management and coordinated multifactorial intervention. Contemporary SGLT2 inhibitor and incretin‐based trials add direct cardiovascular, heart‐failure and kidney outcome evidence in defined populations, while MASH trials provide emerging histological evidence. HbA1c, validated risk equations and organ‐specific pathways retain their established roles.

**Conclusions:**

The proposed framework is interpretive rather than predictive. It does not assign numerical weights or treatment thresholds but aims to identify the preventable outcome that should take priority, the intervention supported by the most relevant evidence and whether that intervention can be implemented safely and durably.

## Graphical abstract

Complication prevention in Type 2 diabetes across the life course. The visual abstract summarises a life‐course approach to complication prevention in Type 2 diabetes. Cumulative metabolic exposure represents the burden carried forward from previous glycaemic, blood‐pressure, lipid and adiposity‐related exposure; organ vulnerability identifies the preventable outcome that has become clinically dominant; and treatment durability reflects whether therapeutic benefit can be realised safely and sustained over time. Organ‐directed therapy should complement, rather than replace, multifactorial risk‐factor management and treatment effects should be interpreted according to molecule‐, population‐ and endpoint‐specific evidence rather than extrapolated uniformly across organs. Clinical prioritisation therefore begins with immediate safety and established organ disease, followed by reduction of residual modifiable risk and attention to durable implementation. The proposed construct is an interpretive framework, not a validated risk score or rigid treatment algorithm. ASCVD, atherosclerotic cardiovascular disease; CKD, chronic kidney disease; CV, cardiovascular; MASH, metabolic dysfunction‐associated steatohepatitis; MASLD, metabolic dysfunction‐associated steatotic liver disease.

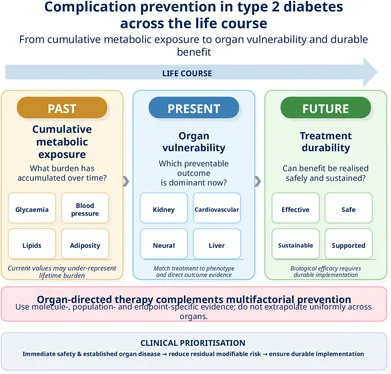

**Abbreviations**
ASCVD: atherosclerotic cardiovascular disease
CKD: chronic kidney disease
eGFR: estimated glomerular filtration rate
HbA1c: glycated haemoglobin
MASH: metabolic dysfunction‐associated steatohepatitis
MASLD: metabolic dysfunction‐associated steatotic liver disease
RECODe: risk equations for complications of Type 2 diabetes
SGLT2: sodium‐glucose cotransporter 2
UKPDS‐OM2: UK Prospective Diabetes Study Outcomes Model 2

## 1 Introduction

Type 2 diabetes is a progressive and heterogeneous multisystem disorder. Its long‐term consequences arise from the interaction of hyperglycaemia, adiposity, haemodynamic stress, dyslipidaemia, inflammation, ageing, behaviour and treatment exposure [1, 2, 3, 4, 5]. Glycated haemoglobin (HbA1c) remains indispensable for assessing average glycaemia, monitoring therapeutic response and defining individualised treatment goals. It should remain central to diabetes care. It should not, however, be interpreted as a complete representation of future complication risk.

The limitation is temporal as well as biological. A current HbA1c value cannot reconstruct the intensity and duration of previous exposure, distinguish recent improvement from consistently favourable control, or identify which organ has already become vulnerable. It also provides limited information on hypoglycaemia, glycaemic variability, treatment workload and the feasibility of sustaining the prescribed regimen. The clinically relevant question is therefore not whether glycaemic control matters, but how it should be interpreted within the wider disease trajectory.

This question has become more important as Type 2 diabetes begins earlier and is lived with for longer. Global prevalence is projected to increase substantially through 2050, while youth‐onset and early‐adult‐onset disease bring kidney, retinal, neurological and cardiovascular complications into working‐age adulthood [6, 7, 8, 9]. Earlier onset extends exposure not only to hyperglycaemia, but also to insulin resistance, obesity‐related inflammation, blood‐pressure burden, atherogenic dyslipidaemia and periods of therapeutic inertia. By contrast, in older adults with frailty, recurrent hypoglycaemia or limited functional reserve, immediate safety may be more important than additional glucose lowering.

The complication profile of diabetes is also changing. Improvements in survival and treatment can reduce selected vascular events while kidney disease, heart failure, neuropathy, frailty and treatment‐related harm remain clinically important [3, 4, 5]. Contemporary prevention must therefore identify the outcome most likely to compromise survival, organ function or independence, select an intervention supported by direct evidence and ensure that the intervention can be maintained for the period in which benefit is expected.

Within this wider model, the present review focuses on three interdependent dimensions: cumulative metabolic exposure, organ vulnerability and treatment durability. These dimensions should not be regarded as clinically equivalent or assigned fixed weights. Their relevance depends on the dominant threat at a particular stage of disease. The review therefore follows a clinically ordered sequence: immediate treatment‐related hazards are considered first, established organ disease and organ‐specific risk next, residual modifiable cardiometabolic exposure thereafter and treatment durability and care‐system capacity as determinants of whether the selected intervention can be implemented and sustained. Figure 1 summarises this hierarchy. The accompanying visual abstract provides a complementary life‐course synthesis of cumulative metabolic exposure, organ vulnerability and treatment durability. The framework is intended to support clinical prioritisation rather than numerical risk prediction.

**FIGURE 1:**
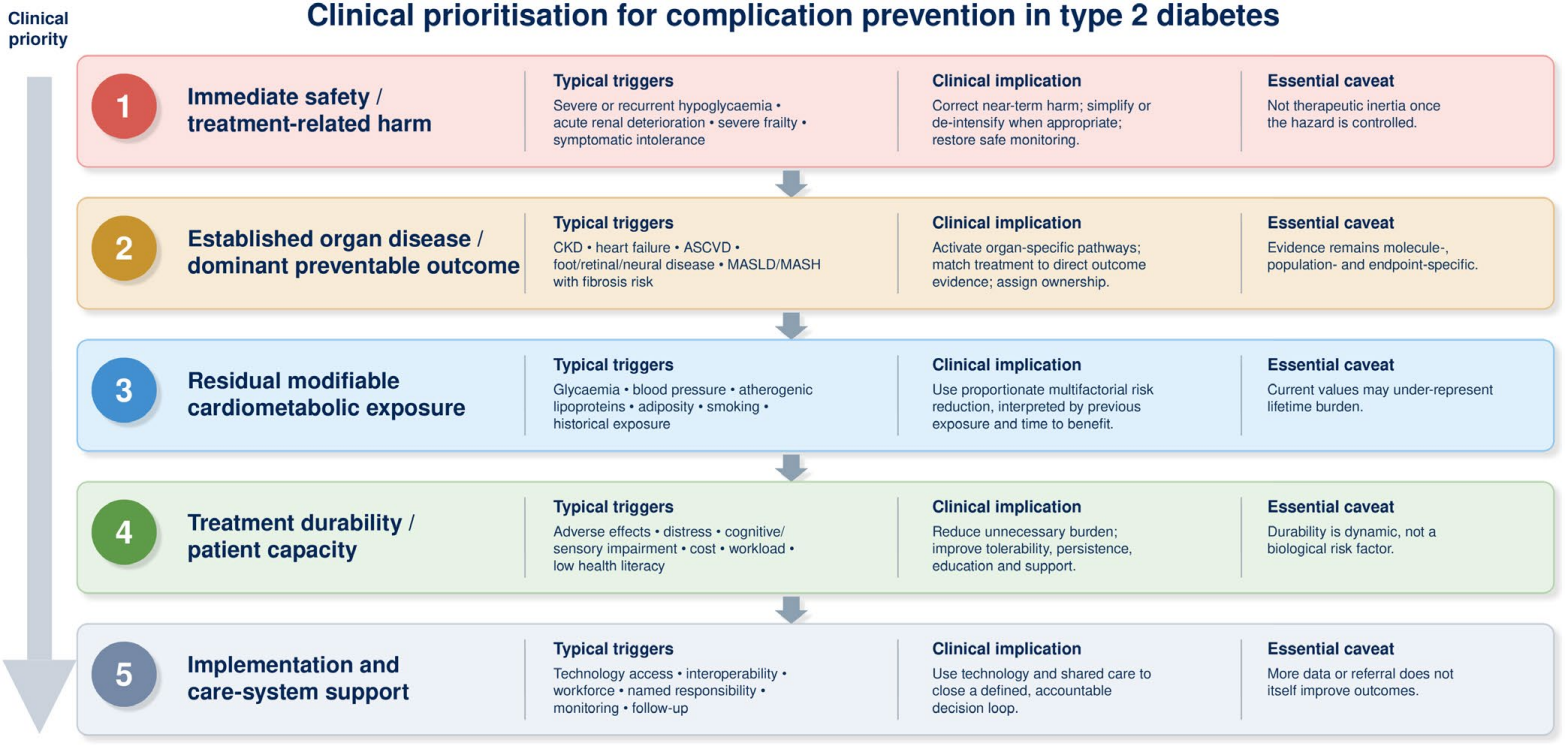
Clinical prioritisation for complication prevention in Type 2 diabetes. The figure presents a contextual hierarchy for clinical review in Type 2 diabetes, prioritising direct outcome evidence where available rather than applying a fixed score. Immediate safety and treatment‐related harm are considered first, followed by established organ disease or a dominant preventable outcome, residual modifiable cardiometabolic exposure, treatment durability and patient capacity, and finally implementation and care‐system support. Each level integrates typical clinical triggers with the corresponding clinical implication and an essential interpretative caveat. The ordering is deliberately contextual and may change according to disease stage, comorbidity, treatment‐related risk, patient capacity and the expected time to benefit. This framework should therefore be interpreted as a clinical hierarchy for prioritisation, not as a validated risk score, fixed weighting system or rigid treatment algorithm. Evidence supporting individual interventions should remain molecule‐, population‐ and endpoint‐specific. ASCVD, atherosclerotic cardiovascular disease; CKD, chronic kidney disease; MASH, metabolic dysfunction‐associated steatohepatitis; MASLD, metabolic dysfunction‐associated steatotic liver disease.

## 2 Methods: Evidence Search and Review Boundaries

This article was developed as a critical narrative review. Targeted searches were conducted in PubMed/MEDLINE through 31 July 2026 and were supplemented by reference‐list screening, international diabetes, cardiovascular, kidney and liver guidance, landmark and contemporary randomised outcome trials, longitudinal cohorts, validated prediction studies and clinically influential reviews. Search concepts combined Type 2 diabetes with age at onset, disease duration, historical glycaemia, metabolic memory, obesity, blood pressure, dyslipidaemia, smoking, multifactorial intervention, complication prediction, albuminuria, kidney‐function decline, atherosclerotic cardiovascular disease, heart failure, neuropathy, foot risk, retinopathy, metabolic dysfunction‐associated steatotic liver disease (MASLD), metabolic dysfunction‐associated steatohepatitis (MASH), hypoglycaemia, continuous glucose monitoring, automated insulin delivery, SGLT2 inhibition, incretin‐based therapy, organ‐protective pharmacotherapy, diabetes distress, treatment burden, health literacy, social determinants, integrated care and health‐system capacity. A targeted update search of these revised domains was completed on 22 September 2026; newly identified publications were screened for material changes to the evidence synthesis, and none required alteration of the principal conclusions.

Priority was given to systematic reviews, meta‐analyses, randomised outcome trials, major long‐term follow‐up studies, large longitudinal cohorts, externally validated models and current international guidance. Historical trials were included to establish how the clinical paradigm evolved, whereas contemporary pharmacotherapy was interpreted at the molecule‐, population‐ and endpoint‐specific level. For MASLD/MASH, histological outcomes were distinguished from liver‐related clinical outcomes. The search was iterative rather than exhaustive. No protocol was registered, no duplicate study selection or formal certainty assessment was undertaken, and no quantitative synthesis was attempted. Supporting Information Table S1 presents the revised search domains and evidence‐selection principles. The manuscript should therefore be read as a clinically oriented critical synthesis, not as a systematic or scoping review.

Four boundaries are maintained throughout. First, the proposed framework does not estimate individual event probabilities, assign numerical weights or define treatment thresholds. Second, the clinical hierarchy described in this review is contextual rather than fixed: immediate safety or established organ disease may dominate management in one individual, whereas cumulative cardiometabolic exposure may remain the principal concern in another. Third, the domains are interdependent rather than mutually exclusive. Earlier exposure influences present organ status, organ dysfunction alters treatment feasibility and treatment durability modifies future exposure. Fourth, psychosocial and health‐system factors are not treated as biological equivalents of albuminuria, heart failure, vascular disease or neuropathy; they are included because they influence whether evidence‐based prevention is accessible, implemented and sustained. The framework should therefore be regarded as an interpretive and hypothesis‐generating structure that complements, but does not replace, validated prediction tools, organ‐specific pathways or individualised clinical judgement.

## 3 From Glycaemic Control to Multifactorial and Organ‐Directed Prevention

HbA1c provides a robust estimate of recent mean glycaemia and remains the principal marker for monitoring long‐term glucose‐lowering treatment. Its value is not in question. The concern arises when a cross‐sectional measure is used to represent a longitudinal and multisystem disease. HbA1c does not identify how long previous glycaemic exposure persisted, whether current improvement occurred before or after organ injury, or whether a favourable average conceals recurrent hypoglycaemia and marked variability.

Glycaemic measures answer related but distinct questions. HbA1c describes average exposure; continuous glucose monitoring characterises time in range, time below range and variability; fasting and postprandial measurements may clarify specific physiological or therapeutic problems [10]. These metrics refine glycaemic assessment and treatment safety, but none independently quantifies kidney, heart‐failure, atherosclerotic, neurological, hepatic or functional risk. Their correct role is to inform glycaemic efficacy and safety within a broader clinical interpretation.

The contemporary approach to complication prevention is best understood as an evolution rather than a replacement of earlier principles. UKPDS 33 established that improved glycaemic control reduces microvascular complications, while UKPDS 38 demonstrated that treatment of coexisting hypertension can influence a broader range of diabetes‐related outcomes [11, 12]. CARDS subsequently reinforced that cardiovascular prevention cannot be organised around glycaemia alone [13]. The Steno‐2 trial then provided a clinically important synthesis: simultaneous attention to glycaemia, blood pressure, lipids, antithrombotic therapy and lifestyle reduced cardiovascular events in a high‐risk population with Type 2 diabetes and microalbuminuria [14]. These studies established the foundations of multifactorial diabetes care (Figure 2). Selected landmark trials informing this transition, together with their principal contribution and interpretative limitations, are summarised in Supporting Information Table S2.

**FIGURE 2:**
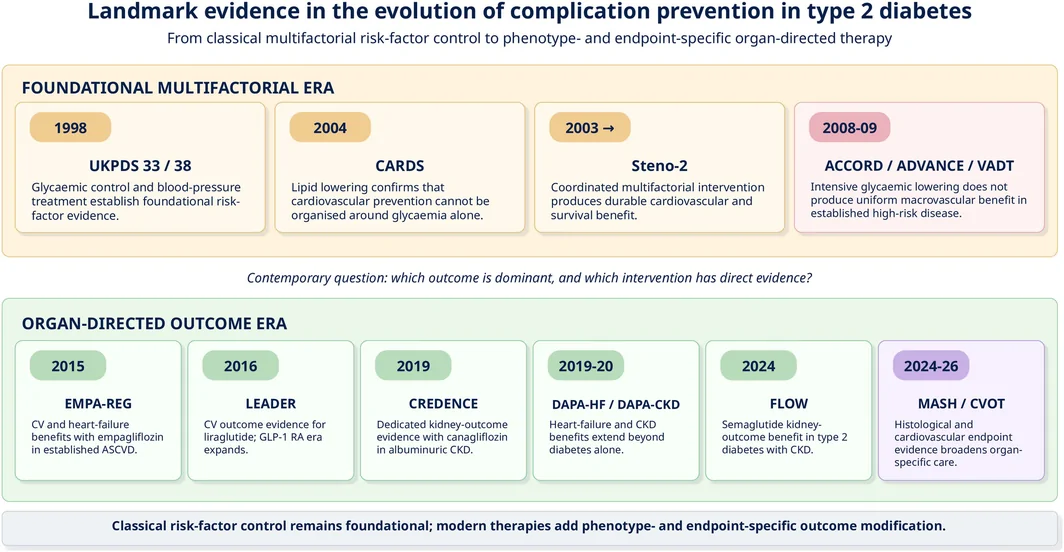
Landmark evidence in the evolution of complication prevention in Type 2 diabetes. The figure illustrates the evolution of complication prevention from classical glycaemic, blood‐pressure and lipid management towards coordinated multifactorial intervention and, subsequently, phenotype‐ and endpoint‐specific organ‐directed therapy. UKPDS 33/38, CARDS and Steno‐2 represent foundational evidence supporting multifactorial risk‐factor management, whereas ACCORD, ADVANCE and VADT illustrate the limits of intensive glycaemic lowering as a uniform strategy for macrovascular risk reduction in people with established or high cardiovascular risk. More recent cardiovascular and organ‐outcome trials, including EMPA‐REG OUTCOME, LEADER, CREDENCE, DAPA‐HF, DAPA‐CKD and FLOW, demonstrate the expansion of treatment selection towards cardiovascular, heart‐failure and kidney outcomes in defined populations. Contemporary MASH and cardiovascular outcome programmes further broaden the organ‐specific evidence base. Classical risk‐factor control therefore remains foundational, while modern therapies add phenotype‐ and endpoint‐specific outcome modification. The timeline is illustrative rather than exhaustive. Trial populations, comparators, endpoints, background therapy and treatment eras differ; effect sizes should therefore not be compared directly across trials. ASCVD, atherosclerotic cardiovascular disease; CKD, chronic kidney disease; CV, cardiovascular; CVOT, cardiovascular outcome trial; GLP‐1 RA, glucagon‐like peptide‐1 receptor agonist; MASH, metabolic dysfunction‐associated steatohepatitis.

The subsequent intensive‐glycaemia trials added an equally important qualification. In ACCORD, a strategy targeting near‐normal HbA1c in a population with established or high cardiovascular risk did not significantly reduce the primary major cardiovascular outcome and was stopped early because mortality was higher in the intensive‐treatment group [15]. ADVANCE achieved a reduction in the combined major vascular outcome that was driven predominantly by microvascular, particularly renal, benefit, without a clear reduction in major macrovascular events [16]. VADT likewise did not demonstrate a significant reduction in major cardiovascular events during the main trial period [17]. These findings do not weaken the importance of glycaemic control; rather, they show that treatment intensity, timing, disease duration, baseline risk and the method by which glycaemia is lowered matter. A lower HbA1c should therefore not be interpreted as a universally dominant therapeutic objective.

The modern therapeutic era has added a further layer. Cardiovascular outcome and dedicated organ‐outcome trials have shown that selected glucose‐lowering agents can modify clinically important cardiovascular, heart‐failure and kidney endpoints in appropriately defined populations. Empagliflozin in EMPA‐REG OUTCOME and liraglutide in LEADER provided early evidence that treatment selection could be informed by cardiovascular outcomes rather than glycaemic efficacy alone [18, 19]. CREDENCE established dedicated renal‐outcome benefit with canagliflozin in Type 2 diabetes with albuminuric chronic kidney disease [20]. DAPA‐HF and DAPA‐CKD extended the clinical relevance of SGLT2 inhibition to heart failure and chronic kidney disease populations that included people without diabetes, reinforcing that these benefits cannot be explained simply by glucose lowering [21, 22]. More recently, FLOW demonstrated kidney‐outcome benefit with semaglutide in people with Type 2 diabetes and chronic kidney disease [23].

This evolution should not be interpreted as a shift from risk‐factor management to drug‐class management. Blood pressure, atherogenic lipoproteins, smoking, adiposity and glycaemic exposure remain major determinants of long‐term outcomes. The contribution of contemporary pharmacotherapy is that, once a clinically relevant organ phenotype is recognised, treatment can increasingly be aligned with direct outcome evidence rather than chosen solely to improve an intermediate metabolic marker. The resulting model is additive: classical multifactorial prevention remains the foundation, while organ‐directed therapies allow priorities to be refined according to the outcome that has become most preventable.

## 4 Cumulative Metabolic Exposure Across the Life Course

Age at onset and disease duration alter the clinical meaning of current control. An individual diagnosed in early adulthood may face several decades of future exposure and substantial lifetime risk even when a conventional short‐term event estimate is modest. Youth‐onset Type 2 diabetes is characterised by early accumulation of kidney, retinal, neurological and cardiovascular complications [8, 9]. In later life, the same glycaemic value may require a different response because frailty, competing illness, hypoglycaemia and a shorter time to benefit change the balance of treatment.

Long‐term follow‐up of the UK Prospective Diabetes Study demonstrated persistent benefit from earlier glycaemic intervention, and analyses of historical HbA1c found that earlier glycaemic exposure was more strongly associated with later outcomes than more recent values [24, 25]. These observations do not justify indiscriminate intensification. They show that recent improvement is clinically valuable but may not fully recover an opportunity lost during earlier years of adverse exposure.

Cumulative exposure is broader than hyperglycaemia. Central adiposity promotes insulin resistance, beta‐cell stress, ectopic lipid deposition and inflammatory signalling [26]. The global rise in obesity indicates that these exposures increasingly begin earlier and persist for longer [27]. Weight management should therefore be considered a central therapeutic component of diabetes care, particularly when sustained weight reduction improves glycaemia, cardiovascular risk factors, physical function and the possibility of simplifying treatment [28].

Blood pressure, atherogenic lipoproteins, smoking, sleep disruption and delayed treatment intensification add vascular and renal stress. Their effects are not identical and should not be combined mechanically. What matters clinically is the duration and clustering of adverse exposure. Social and economic conditions influence whether these abnormalities are corrected or allowed to persist. Medication affordability, food environment, employment, transport, health literacy, continuity and access to preventive care can extend the period during which modifiable risk remains insufficiently treated [29, 30].

Contemporary observational evidence provides a useful counterpoint: excess cardiovascular and mortality risk was progressively lower among people with Type 2 diabetes who had a greater number of major risk factors within target ranges [31]. Long‐term follow‐up of Steno‐2 similarly demonstrates that coordinated multifactorial intervention can produce durable cardiovascular and survival benefit [32, 33]. The lesson is not that every variable should be pursued to the same target in every patient, but that timing, breadth and persistence of risk‐factor control are central to the amount of future risk that remains potentially modifiable.

A life‐course interpretation therefore avoids two opposite errors: false reassurance after recent metabolic improvement in an individual with substantial previous exposure, and unnecessary intensification when treatment‐related harm has become the more immediate threat. Historical exposure establishes the context in which current risk is interpreted, but it does not by itself determine the next clinical action. That decision depends increasingly on whether cumulative exposure has translated into detectable organ vulnerability.

## 5 Organ Vulnerability and Early Complication Signals

The transition from accumulated exposure to clinically manifest risk is neither uniform nor synchronous across organs. Individuals with apparently similar cardiometabolic histories may develop kidney disease, heart failure, atherosclerotic vascular disease, neuropathy, retinal disease or metabolic liver disease at different times and with different rates of progression. Genetic susceptibility, haemodynamic stress, local tissue biology, comorbidity and previous treatment contribute to this heterogeneity. The practical implication is that longitudinal risk assessment should move from asking how much adverse exposure has accumulated to determining which organ or function has become sufficiently vulnerable to change management.

Kidney disease illustrates the distinction between state and direction. Albuminuria may identify renal and vascular vulnerability before advanced filtration loss, while serial estimated glomerular filtration rate (eGFR) measurements can reveal progression that a single value misses [34]. Confirmation and context remain essential because acute illness, haemodynamic change and analytical variation can produce transient abnormalities. Once persistent albuminuria or a declining eGFR trajectory is established, kidney and cardiovascular protection, blood‐pressure control, medication safety and defined surveillance should be addressed explicitly.

Atherosclerotic cardiovascular disease and heart failure similarly change the treatment objective. A validated cardiovascular equation is appropriate when a defined event probability is required, but established heart failure, albuminuric kidney disease and long expected lifetime exposure are not interchangeable risk states. Organ‐specific pathways and outcome trials provide the relevant evidence once the phenotype has been recognised. The integrative task is to ensure that the correct pathway is activated early and maintained.

Retinal disease is another marker of established microvascular vulnerability. Its presence should prompt assessment of the wider complication burden rather than being treated as an isolated specialist finding. The broader principle is that organ status cannot be inferred from the current metabolic snapshot. Existing injury, serial change and functional consequences provide clinically independent information.

Neurological and functional manifestations are less consistently represented in general risk equations. Distal sensorimotor polyneuropathy, autonomic dysfunction, sensory loss, foot deformity and previous ulceration can alter mobility, falls risk, exercise feasibility, treatment safety and self‐management capacity [35, 36]. In a regional clinical cohort, distal sensorimotor polyneuropathy clustered with retinopathy and cardiovascular complications, supporting its interpretation within a broader multisystem burden [37]. Function should therefore be considered an outcome to preserve, not a late consequence to document.

MASLD should also be considered within a multisystem assessment of Type 2 diabetes. The current nomenclature defines MASLD as steatotic liver disease occurring in the presence of cardiometabolic risk factors, and Type 2 diabetes identifies a population at particularly high risk of progressive fibrosis [38, 39]. The clinical issue is therefore not simply the presence of hepatic steatosis, which is common, but the identification of individuals at risk of MASH, advanced fibrosis and subsequent liver‐related complications. Current guidance supports non‐invasive fibrosis risk assessment in people with Type 2 diabetes, with escalation to liver‐stiffness measurement or specialist evaluation when initial risk is elevated.

MASLD is relevant to the present framework for two reasons. First, it reflects cumulative metabolic exposure, particularly adiposity and insulin resistance, while also representing an organ phenotype with its own progression pathway. Second, it can alter treatment priorities because sustained weight reduction and appropriate management of cardiometabolic risk are central to care. Liver disease should therefore neither be treated as an incidental imaging finding nor assumed to carry the same evidentiary structure as diabetic kidney disease or established atherosclerotic cardiovascular disease.

Beyond liver‐related outcomes, MASLD may also mark a broader systemic cardiometabolic phenotype. In Type 2 diabetes, observational evidence suggests associations between MASLD, particularly fibrosis or more advanced disease, and atherosclerotic burden, while meta‐analytic data link NAFLD/MASLD with an increased risk of incident chronic kidney disease; however, shared adiposity, insulin resistance, hypertension, dyslipidaemia and other metabolic exposures limit causal attribution [40, 41]. The relationship with diabetic retinopathy is less consistent: meta‐analytic evidence in Type 2 diabetes has not demonstrated a uniform association between NAFLD and retinopathy, although individual studies suggest that greater fibrosis burden may identify higher‐risk phenotypes [42]. MASLD should therefore be interpreted as part of an interconnected multisystem metabolic phenotype, while cardiovascular, renal and retinal complications continue to require established organ‐specific risk assessment and management.

Glycaemic instability constitutes a further form of vulnerability. Time in range, time below range and variability can reveal unsafe patterns concealed by an average [10]. These measures do not quantify lifetime multisystem risk, but they can identify a need for dose adjustment, regimen simplification, education or closer review. A low HbA1c associated with recurrent hypoglycaemia is not a favourable treatment outcome.

Recognition of organ vulnerability is clinically useful only when it changes the therapeutic objective. Once kidney disease, heart failure, atherosclerotic cardiovascular disease or another clinically important organ phenotype is established, treatment selection should no longer be organised solely around attainment of a glycaemic target. The relevant question becomes which intervention has demonstrated benefit for the outcome that has become dominant, and whether that intervention can be delivered safely and for a sufficient duration to realise that benefit.

## 6 Contemporary Organ‐Protective Pharmacotherapy

Contemporary glucose‐lowering therapy has altered the relationship between metabolic control and complication prevention. Treatment selection is no longer determined only by the magnitude of HbA1c reduction. For selected agents, randomised outcome trials provide direct evidence for cardiovascular, heart‐failure or kidney benefit, allowing therapy to be aligned with a dominant organ phenotype. This development is clinically important because it links treatment choice to outcomes that matter independently of whether additional glucose lowering is required.

The SGLT2 inhibitor evidence is particularly illustrative. EMPA‐REG OUTCOME established cardiovascular benefit with empagliflozin in people with Type 2 diabetes and established atherosclerotic cardiovascular disease, with a prominent reduction in heart‐failure outcomes [18]. CREDENCE subsequently provided dedicated kidney‐outcome evidence for canagliflozin in Type 2 diabetes with albuminuric chronic kidney disease [20]. DAPA‐HF and DAPA‐CKD then demonstrated benefit in heart failure and chronic kidney disease populations that included participants without diabetes [21, 22]. Meta‐analytic evidence supports broader cardiovascular and kidney benefits within studied SGLT2 inhibitor populations, but individual molecules, kidney phenotypes and endpoints should not be treated as interchangeable [43].

Incretin‐based therapy has developed along a partly different pathway. LEADER established a reduction in major cardiovascular events with liraglutide in high‐risk Type 2 diabetes [19], and meta‐analysis of GLP‐1 receptor agonist outcome trials supports cardiovascular benefit across the studied programmes while preserving clinically relevant heterogeneity [44]. FLOW subsequently demonstrated that semaglutide reduced the risk of a major kidney‐disease composite in Type 2 diabetes with chronic kidney disease (hazard ratio 0.76, 95% CI 0.66–0.88) [23]. Evidence is continuing to broaden, but established clinical outcomes should remain distinct from mechanistic or surrogate effects.

This distinction is also important for newer dual‐incretin therapy. In SURPASS‐CVOT, tirzepatide was noninferior to dulaglutide for cardiovascular death, myocardial infarction or stroke, but superiority was not demonstrated (hazard ratio 0.92, 95.3% CI 0.83–1.01) [45]. The result supports cardiovascular efficacy relative to an active GLP‐1 receptor agonist comparator, but should not be represented as proof of greater cardiovascular protection.

The liver domain illustrates both the promise and the limits of an organ‐directed approach. In the Phase 2 SYNERGY‐NASH trial, tirzepatide improved MASH resolution and fibrosis‐related histological endpoints in participants with biopsy‐confirmed MASH and F2‐F3 fibrosis [46]. In the Phase 3 ESSENCE interim analysis, semaglutide 2.4 mg weekly increased MASH resolution without worsening of fibrosis (62.9% vs. 34.3%) and fibrosis improvement without worsening of MASH (36.8% vs. 22.4%) at 72 weeks [47]. These are clinically important histological findings, but they should not yet be interpreted as proof that incretin therapy prevents hepatic decompensation, hepatocellular carcinoma, transplantation or liver‐related mortality.

Current consensus recommendations already place cardiorenal status, weight, hypoglycaemia risk and comorbidity alongside glycaemic efficacy when selecting treatment [48, 49]. The practical consequence is a shift from a glucose‐centred sequence to phenotype‐informed treatment selection. In a patient with chronic kidney disease or heart failure, direct organ‐outcome evidence may justify treatment independent of whether the current HbA1c would otherwise prompt intensification. Conversely, favourable effects on body weight or intermediate biomarkers should not be assumed to prevent every chronic complication. The hierarchy of evidence remains endpoint‐specific: hard cardiovascular, heart‐failure and kidney outcomes should be distinguished from changes in weight, albuminuria, liver histology or other intermediate measures.

Modern pharmacotherapy therefore complements rather than displaces classical multifactorial prevention. Blood‐pressure control, lipid lowering, smoking cessation, weight management and individualised glycaemic treatment remain foundational. The added value of contemporary therapies is that they allow treatment to be matched more directly to established organ vulnerability, provided that the expected benefit is applicable to the patient in front of the clinician and the treatment can be sustained.

## 7 Treatment Durability and Patient Capacity

In diabetes research, treatment durability often refers to persistence of glycaemic efficacy. For complication prevention, the term requires a broader definition: sustained delivery of clinically meaningful metabolic, organ‐protective and safety benefit. It includes biological efficacy, tolerability, persistence, treatment workload, affordability, monitoring, patient capacity and continuity of care.

Treatment initiation is not equivalent to risk modification. An intervention with strong outcome evidence produces little benefit when it is unaffordable, poorly tolerated, misunderstood, intermittently supplied or not reassessed. Diabetes distress, depressive symptoms and treatment fatigue can reduce medication persistence, attendance and willingness to engage with technology [50, 51, 52, 53]. Frailty, cognitive impairment, sensory loss and fear of hypoglycaemia may restrict the complexity of regimens that can be used safely [54, 55].

Durability should be assessed over time rather than recorded as a binary property. Early discontinuation, repeated interruption, progressive regimen complexity and missed surveillance can erode benefit before conventional treatment failure becomes evident. Conversely, a less intensive regimen that is consistently used and regularly reviewed may provide greater long‐term protection than a theoretically superior strategy that cannot be maintained.

Patient capacity is dynamic. It includes health literacy, cognitive and sensory function, social support, financial strain, employment, caregiving responsibilities and the practical ability to complete treatment tasks. Capacity can deteriorate during acute illness, bereavement, job disruption or progression of complications and can improve after education, simplification, psychological care or social support. Pharmacological appropriateness is therefore necessary, but it is not sufficient.

Minimally disruptive medicine provides a useful clinical principle: the preferred regimen is the least burdensome arrangement capable of achieving the outcomes that matter [56, 57]. Treatment matching may require intensification, but it may also require consolidation, deprescribing, shorter educational steps, mental‐health support, medication‐access assistance or clearly defined shared care. Durability is a property of the treatment architecture, not of the molecule alone.

## 8 Technology and Health‐System Capacity as Enabling Components

Diabetes technology has progressed from intermittent point glucose measurements to continuous sensing, automated insulin delivery and integrated decision support. Each technological step can improve the characterisation of glycaemia or the execution of treatment, but also creates new requirements for access, literacy, training, data review and clinical governance. Supporting Information Figure S1 summarises this progression and its principal limitations.

Continuous glucose monitoring can characterise time in range, variability and hypoglycaemia, and randomised evidence supports its use in selected adults with Type 2 diabetes treated with basal insulin [58, 59]. Automated insulin delivery can improve glycaemic outcomes in selected insulin‐treated populations, although benefit remains dependent on sustained device use, education and clinical support [59, 60]. These technologies improve measurement or treatment execution; they should not be interpreted as independent measures of lifetime multisystem risk.

Digital interventions and telemedicine can improve glycaemic outcomes and continuity, but their effects vary according to design, uptake, population and health‐system context [61, 62, 63]. The central limitation is increasingly not data generation but data translation. A report has clinical value when it changes medication, education, simplification, hypoglycaemia prevention, follow‐up or organ‐risk management. More data without a responsible response increase documentation rather than benefit.

Complication prevention is distributed across primary care, diabetology, cardiology, nephrology, ophthalmology, neurology, podiatry, hepatology and mental‐health services. This breadth reflects the biology of Type 2 diabetes, but it also creates opportunities for fragmentation. Screening should produce an interpretable result; the result should trigger a defined response; treatment should have a responsible prescriber; safety monitoring should be assigned; and the response should be reviewed at an agreed interval.

Care‐system durability therefore requires access, interoperability, workforce capacity and governance. Shared care should be defined by tasks rather than institutional boundaries. Referral without an interim plan, or a specialist recommendation without transfer of responsibility, leaves the complication pathway insufficiently managed. Equity is integral to effectiveness: digital exclusion, medication cost, transport, language, limited health literacy and uneven specialist availability can influence access to early organ‐protective care and the persistence of undertreated risk [29, 30]. Technology and care systems are consequently enabling components of prevention, not co‐equal biological risk factors.

## 9 Relationship to Established Risk Assessment and Precision Diabetes Care

Modern diabetes care already includes robust methods of prediction and stratification. SCORE2‐Diabetes estimates 10‐year cardiovascular risk in European populations, while the UKPDS Outcomes Model 2 and RECODe estimate selected macrovascular, microvascular and mortality outcomes [64, 65, 66]. These tools should be used for the endpoints, populations and time horizons for which they were developed.

Risk models have boundaries related to population, treatment era, endpoint definition and calibration. External validation of RECODe and UKPDS‐OM2 has shown that performance can vary across cohorts and settings [67, 68]. Contemporary therapies alter event rates and competing risks, reinforcing the need for recalibration and local validation. A qualitative framework cannot quantify risk more accurately than a validated model and should not be used to create untested thresholds.

The contribution proposed here is integration rather than replacement. A cardiovascular estimate, albuminuria, recurrent nocturnal hypoglycaemia, clinically significant liver fibrosis risk, treatment distress and difficulty obtaining medication may each be valid findings, yet remain clinically fragmented. The relevant question is whether their combination changes the dominant preventable outcome and the action required now. Table 1 compares established assessment approaches with this complementary clinical purpose.

**TABLE 1:** Established assessment approaches and the complementary contribution of an integrative life‐course review.

Approach	Primary contribution	What may remain outside its purpose	Complementary clinical question
Current glycaemic assessment	Measures recent mean glycaemia and treatment response; CGM adds variability and hypoglycaemia information	Historical exposure, multisystem vulnerability and feasibility of sustained treatment	Is the current pattern representative of a stable trajectory, and does it conceal safety risk?
Cardiovascular risk equations	Estimate defined cardiovascular endpoints over a specified horizon	Some organ‐specific, functional and implementation domains; lifetime exposure beyond the model horizon	Would age at onset, organ disease or treatment capacity change action despite the estimated short‐term risk?
Diabetes outcome models	Estimate selected macrovascular, microvascular and mortality outcomes	Outcomes and variables not included in the model; treatment‐era and calibration differences	Which clinically important dimension is absent, and would recognising it alter management now?
Organ‐specific pathways	Link CKD, ASCVD, heart‐failure and relevant liver phenotypes to evidence‐based treatment and monitoring	Integration across organs, historical exposure, workload and care ownership	Is the pathway being implemented early, durably and with explicit responsibility?
Integrative life‐course review	Interprets exposure, organ vulnerability, treatment durability, technology and care delivery together	Not validated for numerical prediction and should not define treatment thresholds	Which preventable outcome is dominant, what is the least burdensome effective response, and who will reassess it?

Type 2 diabetes is heterogeneous, and data‐driven subgroups have strengthened interest in phenotype‐directed care [69]. The present framework is deliberately dynamic rather than diagnostic. The dominant vulnerability may change after new kidney disease, heart failure, metabolic liver disease, weight gain, frailty, loss of self‐management support or treatment intolerance. The most appropriate regimen is therefore not permanently defined at diagnosis. It is the least burdensome effective strategy that continues to address the outcome most likely to compromise survival, organ function or independence.

## 10 Clinical Translation: from Recognition to Accountable Action

Clinical integration requires prioritisation rather than accumulation of information. Figure 1, Table 2 summarise a contextual hierarchy for this purpose. The first question is whether an immediate treatment‐related or metabolic hazard requires action. Recurrent or severe hypoglycaemia, acute deterioration in kidney function, severe frailty, symptomatic treatment intolerance or inability to manage a complex regimen may take precedence over further long‐term risk‐factor intensification because they can produce near‐term harm.

**TABLE 2:** Clinical priority hierarchy for complication prevention in Type 2 diabetes.

Priority level	Typical triggers	Why this may take precedence	Clinical implication	Essential caveat
1. Immediate safety/treatment‐related harm	Recurrent or severe hypoglycaemia; acute deterioration in kidney function; severe frailty; symptomatic intolerance; inability to manage a complex regimen	Near‐term preventable harm may outweigh the expected benefit of further long‐term intensification	Correct the safety problem; simplify or de‐intensify where appropriate; review acute contributors and monitoring needs	This is not a mandate for therapeutic inertia once the immediate hazard has been addressed
2. Established organ disease or dominant preventable outcome	CKD; heart failure; ASCVD; active foot disease; advanced retinal/neurological complications; MASLD/MASH with clinically significant fibrosis risk	Direct outcome evidence or organ‐specific pathways may change treatment priority independently of current HbA1c	Activate organ‐specific guideline‐directed treatment and surveillance; define ownership and time to reassessment	Organ phenotypes are heterogeneous; evidence is molecule‐, population‐ and endpoint‐specific
3. Residual modifiable cardiometabolic exposure	Glycaemia; blood pressure; atherogenic lipoproteins; adiposity; smoking; persistent historical exposure	These remain major determinants of long‐term vascular and microvascular risk	Use proportionate multifactorial risk reduction, interpreted in the context of previous exposure and time to benefit	Current values may under‐represent cumulative exposure; more intensive treatment is not uniformly beneficial in every patient
4. Treatment durability/patient capacity	Adverse effects; treatment fatigue; distress; cognition/sensory impairment; polypharmacy; cost; low health literacy	Biological efficacy cannot become realised benefit when treatment cannot be sustained safely	Reduce unnecessary workload; improve tolerability, persistence, education and support	Durability is dynamic and context‐dependent; it is not a biological risk factor equivalent to organ disease
5. Enabling care systems	Technology access; interoperability; workforce; named responsibility; monitoring; follow‐up	These influence whether risk recognition is translated into timely and accountable care	Use technology and shared care to close a defined decision loop	More data, referral or technological complexity does not by itself improve clinical outcomes

*Note:* The hierarchy is contextual and non‐validated. It orders clinical questions and does not assign fixed numerical weights or treatment thresholds.

In clinically stable patients, the next priority is established organ disease or a clinically meaningful organ‐specific risk state. Chronic kidney disease, heart failure, atherosclerotic cardiovascular disease, active diabetic foot disease, advanced retinal or neurological complications or MASLD/MASH with clinically significant fibrosis risk should direct attention towards organ‐specific management and surveillance, prioritising interventions supported by direct clinical outcome evidence where such evidence is available. In this setting, the objective is not to optimise every measurable variable simultaneously, but to identify the outcome most likely to compromise survival, organ function or independence and to address it without unnecessary delay.

Residual modifiable exposure should then be reviewed comprehensively. Glycaemia, blood pressure, atherogenic lipoproteins, adiposity and smoking remain central determinants of long‐term risk, but the intensity and sequence of intervention should reflect baseline risk, previous exposure, competing morbidity and the expected time to benefit. Recent improvement in one domain should not provide false reassurance when substantial exposure or established organ injury persists.

Treatment durability forms a further level of clinical assessment rather than a competing biological risk factor. A pharmacologically appropriate regimen cannot deliver its expected benefit when adverse effects, hypoglycaemia, treatment workload, cognitive or sensory impairment, cost, limited health literacy or fragmented follow‐up prevent sustained use. Technology, integrated care and decision support should similarly be regarded as enabling components: their clinical value lies in improving recognition, implementation, safety and reassessment.

The resulting hierarchy is deliberately contextual rather than numerical. It does not assign fixed weights to competing risks and should not replace validated cardiovascular or organ‐specific prediction tools. Its purpose is to ensure that the most consequential and modifiable problem is addressed first, while lower‐priority domains remain subject to longitudinal review. The review is incomplete until responsibility is explicit: the selected action, the person or service responsible for it, the monitoring plan and the timing of reassessment should all be defined.

## 11 Research Agenda

The integrated framework should remain hypothesis‐generating until formally developed. A first step is consensus‐based specification of a minimum clinically meaningful set of historical exposure, organ, capacity, technology and care‐delivery domains. Development should include diabetology, primary care, cardiology, nephrology, hepatology, neurology, psychology, nursing, implementation science and people living with diabetes.

Retrospective longitudinal studies should determine whether serial exposure measures, early organ signals and indicators of treatment persistence or capacity add useful information to established models. Evaluation should be outcome‐specific and include discrimination, calibration, reclassification and decision‐curve analysis where appropriate. Additional complexity without a change in clinical action should not be considered progress.

External validation is required across age groups, ethnic backgrounds, health systems and treatment environments. Feasibility, missingness, measurement burden and equity should be assessed alongside predictive performance. A framework that identifies vulnerability but depends on inaccessible interventions may widen disparities.

Pragmatic clinical‐utility studies could test whether a structured review changes treatment alignment, time to organ‐protective therapy, hypoglycaemia, completion of surveillance, patient‐reported workload or continuity of responsibility. Cluster‐randomised or stepped‐wedge designs may be appropriate because the intervention contains interacting clinical and organisational components [70, 71]. The decisive outcome is not conceptual agreement, but earlier, safer and more durable care.

## 12 Limitations

This review is a targeted narrative synthesis rather than a systematic review. Source selection was guided by clinical relevance, and no formal certainty assessment was performed. Evidence is strongest for individual therapies, validated risk models and organ‐specific outcomes; it is less direct for an integrated life‐course construct.

The landmark‐trial timeline is a narrative device rather than a systematic chronology of all influential trials. Studies were selected because they illustrate major transitions in the clinical logic of complication prevention, and their inclusion should not be interpreted as a formal ranking of evidentiary importance. Cross‐trial comparisons are limited by differences in population, baseline risk, treatment era, comparator, follow‐up and endpoint definition.

The proposed clinical hierarchy is contextual and has not been prospectively validated as an algorithm. Historical exposure is often incomplete, organ signals may represent both predictors and established complications and treatment burden may be both a consequence and a cause of worsening control. These relationships make simple additive scoring inappropriate without empirical development.

Treatment durability is intentionally defined more broadly than persistence of glycaemic efficacy and will require further consensus. Patient capacity and care‐system factors are context‐dependent. Recommendations concerning therapy, monitoring, technology and referral must remain aligned with local guidance, resources and professional competence.

Liver evidence requires particular caution. Recent incretin‐based MASH trials have demonstrated histological improvement, whereas effects on hepatic decompensation, hepatocellular carcinoma, transplantation and liver‐related mortality remain to be established. More broadly, cardiovascular, kidney, heart‐failure and liver evidence derives from different trial populations, comparators and endpoints; benefits should therefore be interpreted at the molecule‐, population‐ and endpoint‐specific level rather than extrapolated as uniform class‐wide or pan‐organ effects.

## 13 Conclusions

Glycaemic control remains fundamental to Type 2 diabetes care, but durable complication prevention requires a broader longitudinal interpretation. Cumulative metabolic exposure defines the burden carried forward, organ vulnerability identifies the outcome that has become clinically dominant and treatment durability determines whether evidence‐based intervention becomes sustained benefit.

The historical evidence base supports comprehensive control of glycaemia, blood pressure, atherogenic lipoproteins and other modifiable exposures. Contemporary SGLT2 inhibitor and incretin‐based outcome trials add an organ‐directed dimension to this foundation, allowing selected cardiovascular, heart‐failure and kidney phenotypes to be treated according to direct outcome evidence. Emerging MASH data extend this multisystem perspective, but histological improvement should not be conflated with established liver‐related clinical outcome reduction.

These dimensions should be interpreted through a contextual clinical hierarchy. Immediate treatment‐related hazards may take precedence over further intensification; established organ disease may redirect therapy independently of current HbA1c; residual modifiable exposure remains a core target; and treatment durability and enabling care systems determine whether biological efficacy becomes realised benefit. This ordering is interpretive, not a validated score or treatment algorithm.

The proposed framework does not replace validated risk equations, organ‐specific pathways or individualised glycaemic targets. It provides a structured basis for recognising when fragmented information should prompt re‐prioritisation, simplification, broader risk reduction or clearer ownership of care. Its future value will depend on empirical evidence of feasibility, incremental clinical utility, equity and effects on outcomes that matter to people living with diabetes.

## Author Contributions

**Ferenc Sztanek:** conceptualization, writing – original draft, writing – review and editing, visualization.

## Funding

This work was supported by the Hungarian Diabetes Association. The funder had no role in the conception, evidence synthesis, interpretation, writing or decision to submit the manuscript.

## Ethics Statement

The author has nothing to report.

## Conflicts of Interest

The author declares no conflicts of interest.

## Supporting information


**Supporting Information: Figure S1.** From glucose monitoring to decision support: clinical value and limitations of diabetes technology. The figure illustrates the progression from self‐monitoring of blood glucose through continuous glucose monitoring and automated insulin delivery to data integration and clinical decision support. Increasing technological capability can improve the temporal characterisation of glycaemia, detection of variability and hypoglycaemia, treatment automation, risk stratification and workflow support. These potential benefits are accompanied by increasing data volume and interpretative complexity, including data and device burden, unequal access, algorithmic opacity and the risk of false precision or over‐reliance on automated outputs. The clinical value of each technology therefore depends not only on technical performance, but also on whether the information generated leads to a defined and appropriate clinical action. Real‐world effectiveness is further modified by treatment adherence, psychosocial burden, digital health literacy and health‐system capacity. Technology should consequently be regarded as an enabling component of diabetes care rather than as a substitute for clinical interpretation, individualised treatment decisions or accountable follow‐up. AID, automated insulin delivery; CGM, continuous glucose monitoring; SMBG, self‐monitoring of blood glucose.

**Supporting Information: Table S1.** Revised search domains and evidence‐selection principles.

**Supporting Information: Table S2.** Selected landmark trials informing the evolution of complication prevention.

## Data Availability

No new datasets were generated or analysed. Search concepts and evidence‐selection principles are provided in Supporting Information Table S1; selected landmark evidence informing Figure 2 is summarised in Supporting Information Table S2.
