# Supplementary material for: Complication Prevention Beyond Glycaemic Control in Type 2 Diabetes: Cumulative Metabolic Exposure, Organ Vulnerability and Treatment Durability
Source: Endocrinol Diabetes Metab. 2026 Nov 3;9(6):e70383. doi: 10.1002/edm2.70383 (PMC13731741; doi:10.1002/edm2.70383)
Supplement: Supplementary file 1 [file EDM2-9-e70383-s002.doc]

# Supporting Information Table S1. Revised search domains and evidence-selection principles.

## The table summarises the targeted search domains, priority evidence types and revision-specific selection principles used in this critical narrative review.

Search update: A targeted PubMed/MEDLINE update of the revised evidence domains was completed on 22 September 2026. Newly identified publications were screened for material changes to the evidence synthesis; none required alteration of the principal conclusions.

| **Domain** | **Illustrative concepts / terms** | **Priority evidence** | **Revision-specific purpose** |
| --- | --- | --- | --- |
| Foundational risk-factor evidence | UKPDS; intensive glycaemic control; blood-pressure control; statin; lipid lowering; Steno-2; multifactorial intervention | Landmark randomised trials and long-term follow-up | Establish the historical evidence architecture |
| Balance / limits of intensive glycaemia | ACCORD; ADVANCE; VADT; hypoglycaemia; mortality; macrovascular outcomes; time to benefit | Large randomised outcome trials | Prevent one-sided interpretation of glycaemic intensification |
| Cumulative metabolic exposure | Age at onset; disease duration; historical HbA1c; metabolic memory; obesity; blood pressure; dyslipidaemia; smoking; therapeutic inertia | Long-term trials, longitudinal cohorts, epidemiological studies | Retain the life-course construct while reducing list-like presentation |
| Organ vulnerability | CKD; albuminuria; eGFR decline; ASCVD; heart failure; neuropathy; foot disease; retinopathy; MASLD; MASH; fibrosis | Organ-specific guidelines, outcome trials, clinical reviews | Expand the multisystem model to include metabolic liver disease |
| SGLT2 inhibitor outcomes | EMPA-REG; CREDENCE; DAPA-HF; DAPA-CKD; kidney outcomes; heart-failure outcomes | Randomised cardiovascular, kidney and heart-failure outcome trials | Define organ-specific outcome evidence beyond HbA1c reduction |
| Incretin-based outcomes | LEADER; FLOW; semaglutide; tirzepatide; SURPASS-CVOT; cardiovascular outcomes; kidney outcomes | Randomised cardiovascular and kidney outcome trials | Expand outcome-aligned treatment while preserving molecule/endpoint specificity |
| Metabolic liver disease | MASLD; MASH; fibrosis; FIB-4; liver stiffness; SYNERGY-NASH; ESSENCE | Multisociety nomenclature, EASL-EASD-EASO guidance, randomised MASH trials | Address the reviewer-identified omission and distinguish histology from hard liver outcomes |
| Treatment durability / capacity | Persistence; adherence; tolerability; treatment burden; distress; depression; cognition; health literacy; affordability | Psychosocial research, patient-centred care literature, older-adult guidance | Retain as determinant of realised benefit, not as a biological risk factor |
| Technology / health systems | CGM; AID; decision support; interoperability; access; workforce; shared care; governance | Randomised technology trials, systematic reviews, implementation literature | Reposition as an enabling/implementation domain rather than a co-equal biological risk domain |

## Selection principles

• Priority was given to randomised outcome trials, major long-term follow-up studies, externally validated risk models, systematic reviews/meta-analyses and current international guidance.

• Historical trials were included to establish how the clinical paradigm evolved; their treatment targets were not imported uncritically into current practice.

• Modern pharmacotherapy was described at the molecule-, population- and endpoint-specific level; class-wide or pan-organ claims were avoided unless directly supported.

• For MASLD/MASH, histological endpoints were distinguished from liver-related clinical outcomes.

• No protocol registration, duplicate screening, formal risk-of-bias assessment or quantitative synthesis was undertaken.
