# Supplementary material for: Complication Prevention Beyond Glycaemic Control in Type 2 Diabetes: Cumulative Metabolic Exposure, Organ Vulnerability and Treatment Durability
Source: Endocrinol Diabetes Metab. 2026 Nov 3;9(6):e70383. doi: 10.1002/edm2.70383 (PMC13731741; doi:10.1002/edm2.70383)
Supplement: Supplementary file 2 [file EDM2-9-e70383-s003.doc]

# Supporting Information Table S2. Selected landmark trials informing the evolution of complication prevention.

## The table summarises the selected landmark trials underlying Figure 2, together with their contribution to the review and principal interpretative limitations. It is an illustrative narrative evidence table and not a systematic review or cross-trial efficacy ranking.

| **Trial (year)** | **Domain** | **Population** | **Comparison** | **Contribution to the review** | **Key interpretive limitation** | **Ref.** |
| --- | --- | --- | --- | --- | --- | --- |
| UKPDS 33 (1998) | Glycaemia | Newly diagnosed type 2 diabetes | Intensive sulphonylurea/insulin strategy vs conventional treatment | Reduced aggregate diabetes-related outcomes, driven mainly by fewer microvascular endpoints. | Did not establish macrovascular benefit from intensive glycaemic control; more hypoglycaemia and weight gain. | 11 |
| UKPDS 38 (1998) | Blood pressure | Hypertensive participants with type 2 diabetes | Tighter vs less tight blood-pressure control | Reduced diabetes-related endpoints, stroke and microvascular outcomes. | Targets and background therapy reflect an earlier treatment era; not a direct template for current BP thresholds. | 12 |
| Steno-2 (2003) | Multifactorial risk | Type 2 diabetes with microalbuminuria | Intensive multifactorial vs conventional treatment | Established the value of coordinated management of multiple modifiable risk factors. | Small, selected high-risk cohort; intervention bundled several components and cannot attribute benefit to one factor. | 14 |
| CARDS (2004) | Lipids | Type 2 diabetes without high LDL requirement or established CVD | Atorvastatin vs placebo | Demonstrated cardiovascular event reduction with statin primary prevention in type 2 diabetes. | Trial population and statin intensity differ from contemporary lipid-lowering strategies. | 13 |
| ACCORD (2008) | Glycaemia - balance | Longer-standing, high cardiovascular-risk type 2 diabetes | Very intensive vs standard glycaemic strategy | No significant reduction in the primary major cardiovascular outcome; intensive arm stopped early for excess mortality. | Should not be generalised to all glycaemic lowering or to contemporary agents with independent outcome benefits. | 15 |
| ADVANCE (2008) | Glycaemia - balance | Type 2 diabetes at vascular risk | Intensive gliclazide-based strategy vs standard control | Reduced combined major vascular outcome largely through nephropathy/microvascular benefit; no clear macrovascular reduction. | Effect reflects a specific strategy and population; does not imply that lower HbA1c is uniformly beneficial or harmful. | 16 |
| VADT (2009) | Glycaemia - balance | Veterans with long-standing, suboptimally controlled type 2 diabetes | Intensive vs standard glucose control | No significant reduction in major cardiovascular events during the main trial period. | Population was predominantly male with long diabetes duration; later follow-up does not convert the trial into a universal treatment rule. | 17 |
| EMPA-REG OUTCOME (2015) | SGLT2 / cardiovascular | Type 2 diabetes with established ASCVD | Empagliflozin vs placebo | Established cardiovascular outcome benefit and a prominent heart-failure signal for SGLT2 inhibition. | Single molecule in a secondary-prevention population; individual endpoints and class extrapolation require care. | 18 |
| LEADER (2016) | GLP-1 RA / cardiovascular | Type 2 diabetes at high cardiovascular risk | Liraglutide vs placebo | Reduced major cardiovascular events and established outcome benefit for a GLP-1 receptor agonist. | Does not establish identical cardiovascular effects for all incretin-based therapies. | 19 |
| CREDENCE (2019) | SGLT2 / kidney | Type 2 diabetes with albuminuric CKD | Canagliflozin vs placebo | Provided dedicated renal-outcome evidence in diabetic kidney disease. | Albuminuric CKD population; results should not be extrapolated without regard to kidney phenotype or molecule-specific evidence. | 20 |
| DAPA-HF (2019) | SGLT2 / heart failure | HFrEF with or without type 2 diabetes | Dapagliflozin vs placebo | Showed heart-failure benefit in a population that included people without diabetes, supporting effects beyond glucose lowering. | Not a diabetes-specific trial; the result supports organ-level efficacy, not a claim of universal pan-organ protection. | 21 |
| DAPA-CKD (2020) | SGLT2 / kidney | CKD with or without type 2 diabetes | Dapagliflozin vs placebo | Extended kidney protection to CKD populations with and without diabetes. | Eligibility required defined CKD criteria; cross-trial comparison with diabetic CKD trials is inappropriate. | 22 |
| FLOW (2024) | GLP-1 RA / kidney | Type 2 diabetes with CKD | Semaglutide 1.0 mg weekly vs placebo | Reduced the primary composite of major kidney disease events or cardiovascular death (HR 0.76, 95% CI 0.66-0.88). | Applies to the FLOW CKD population and semaglutide regimen; not a class-wide renal effect. | 23 |
| SYNERGY-NASH (2024) | Dual incretin / liver | Biopsy-confirmed MASH with F2-F3 fibrosis | Tirzepatide vs placebo | Improved MASH resolution and fibrosis-related histological endpoints over 52 weeks. | Phase 2; histological endpoints, limited duration, and no established reduction in liver-related clinical events. | 43 |
| ESSENCE (2025) | GLP-1 RA / liver | Biopsy-confirmed MASH with F2-F3 fibrosis | Semaglutide 2.4 mg weekly vs placebo | At week 72, improved MASH resolution without fibrosis worsening (62.9% vs 34.3%) and fibrosis improvement without MASH worsening (36.8% vs 22.4%). | Interim histological analysis of an ongoing trial; hard liver outcomes and long-term clinical benefit remain to be established. | 44 |
| SURPASS-CVOT (2025) | Dual incretin / cardiovascular | Type 2 diabetes with established ASCVD | Tirzepatide vs dulaglutide | Tirzepatide was noninferior, but not superior, to dulaglutide for cardiovascular death, myocardial infarction or stroke (HR 0.92, 95.3% CI 0.83-1.01). | Active comparator already has cardiovascular benefit; trial does not support superiority or a blanket claim of greater cardiovascular protection. | 42 |

## Reference key

11. UK Prospective Diabetes Study (UKPDS) Group. Intensive blood-glucose control with sulphonylureas or insulin compared with conventional treatment and risk of complications in patients with type 2 diabetes (UKPDS 33). Lancet. 1998;352:837-853. doi:10.1016/S0140-6736(98)07019-6.

12. UK Prospective Diabetes Study Group. Tight blood pressure control and risk of macrovascular and microvascular complications in type 2 diabetes: UKPDS 38. BMJ. 1998;317:703-713. doi:10.1136/bmj.317.7160.703.

14. Gaede P, Vedel P, Larsen N, Jensen GVH, Parving HH, Pedersen O. Multifactorial intervention and cardiovascular disease in patients with type 2 diabetes. N Engl J Med. 2003;348:383-393. doi:10.1056/NEJMoa021778.

13. Colhoun HM, Betteridge DJ, Durrington PN, et al.; CARDS investigators. Primary prevention of cardiovascular disease with atorvastatin in type 2 diabetes in the Collaborative Atorvastatin Diabetes Study (CARDS): multicentre randomised placebo-controlled trial. Lancet. 2004;364:685-696. doi:10.1016/S0140-6736(04)16895-5.

15. Action to Control Cardiovascular Risk in Diabetes Study Group, Gerstein HC, Miller ME, et al. Effects of intensive glucose lowering in type 2 diabetes. N Engl J Med. 2008;358:2545-2559. doi:10.1056/NEJMoa0802743.

16. ADVANCE Collaborative Group, Patel A, MacMahon S, et al. Intensive blood glucose control and vascular outcomes in patients with type 2 diabetes. N Engl J Med. 2008;358:2560-2572. doi:10.1056/NEJMoa0802987.

17. Duckworth W, Abraira C, Moritz T, et al.; VADT Investigators. Glucose control and vascular complications in veterans with type 2 diabetes. N Engl J Med. 2009;360:129-139. doi:10.1056/NEJMoa0808431.

18. Zinman B, Wanner C, Lachin JM, et al.; EMPA-REG OUTCOME Investigators. Empagliflozin, cardiovascular outcomes, and mortality in type 2 diabetes. N Engl J Med. 2015;373:2117-2128. doi:10.1056/NEJMoa1504720.

19. Marso SP, Daniels GH, Brown-Frandsen K, et al.; LEADER Steering Committee; LEADER Trial Investigators. Liraglutide and cardiovascular outcomes in type 2 diabetes. N Engl J Med. 2016;375:311-322. doi:10.1056/NEJMoa1603827.

20. Perkovic V, Jardine MJ, Neal B, et al.; CREDENCE Trial Investigators. Canagliflozin and renal outcomes in type 2 diabetes and nephropathy. N Engl J Med. 2019;380:2295-2306. doi:10.1056/NEJMoa1811744.

21. McMurray JJV, Solomon SD, Inzucchi SE, et al.; DAPA-HF Trial Committees and Investigators. Dapagliflozin in patients with heart failure and reduced ejection fraction. N Engl J Med. 2019;381:1995-2008. doi:10.1056/NEJMoa1911303.

22. Heerspink HJL, Stefánsson BV, Correa-Rotter R, et al.; DAPA-CKD Trial Committees and Investigators. Dapagliflozin in patients with chronic kidney disease. N Engl J Med. 2020;383:1436-1446. doi:10.1056/NEJMoa2024816.

23. Perkovic V, Tuttle KR, Rossing P, et al. Effects of semaglutide on chronic kidney disease in patients with type 2 diabetes. N Engl J Med. 2024;391:109-121. doi:10.1056/NEJMoa2403347.

43. Loomba R, Hartman ML, Lawitz EJ, et al.; SYNERGY-NASH Investigators. Tirzepatide for metabolic dysfunction-associated steatohepatitis with liver fibrosis. N Engl J Med. 2024;391:299-310. doi:10.1056/NEJMoa2401943.

44. Sanyal AJ, Newsome PN, Kliers I, et al.; ESSENCE Study Group. Phase 3 trial of semaglutide in metabolic dysfunction-associated steatohepatitis. N Engl J Med. 2025;392:2089-2099. doi:10.1056/NEJMoa2413258.

42. Nicholls SJ, Pavo I, Bhatt DL, et al.; SURPASS-CVOT Investigators. Cardiovascular outcomes with tirzepatide versus dulaglutide in type 2 diabetes. N Engl J Med. 2025;393:2409-2420. doi:10.1056/NEJMoa2505928.
